# The Absorb bioresorbable vascular scaffold in real-world practice: long-term follow-up of the AMC Single Centre Real World PCI Registry

**DOI:** 10.1007/s12471-019-01362-4

**Published:** 2020-01-17

**Authors:** R. Y. G. Tijssen, M. E. Annink, R. P. Kraak, K. T. Koch, J. Baan Jr, M. M. Vis, J. J. Piek, J. P. S. Henriques, R. J. de Winter, M. A. M. Beijk, J. J. Wykrzykowska

**Affiliations:** 1 Department of Clinical and Experimental Cardiology, Amsterdam Cardiovascular Sciences, Amsterdam UMC, Heart Centre, University of Amsterdam, Amsterdam, The Netherlands; 2 Department of Cardiology, Onze Lieve Vrouwe Gasthuis, Amsterdam, The Netherlands

**Keywords:** Bioresorbable scaffolds, Target-vessel failure, Scaffold thrombosis

## Abstract

**Background:**

Bioresorbable scaffolds have been introduced to overcome the shortcomings of drug-eluting stents. Higher rates of device thrombosis, however, have been reported up to 3 years after implantation of the Absorb bioresorbable vascular scaffold (BVS). In the current article, we therefore report long-term clinical outcomes of the AMC Absorb Registry.

**Methods and results:**

In the AMC Absorb Registry, all patients who underwent a percutaneous coronary intervention with Absorb BVS implantation between 30 August 2012 and 5 August 2013 at the Amsterdam University Medical Centre—Academic Medical Centre were included. The composite endpoint of this analysis was target-vessel failure (TVF). The median follow-up of the study cohort of the AMC Absorb Registry was 1534 days. At the time of the cross-sectional data sweep the clinical status at 4 years was known in 124 of 135 patients (91.9%). At long-term follow-up, the composite endpoint of TVF had occurred in 27 patients. The 4‑year Kaplan-Meier estimate of TVF was 19.8%. At 4 years cardiac death had occurred in 4 patients (3.2%) and target-vessel myocardial infarction in 9 (6.9%) patients. Definite scaffold thrombosis occurred in 5 (3.8%) patients. We found 1 case of very late scaffold thrombosis that occurred at 911 days after device implantation in a patient who was not on dual anti-platelet therapy.

**Conclusion:**

In a patient population reflecting routine clinical practice, we found that cases of TVF continued to accrue beyond 2 years after Absorb BVS implantation.

## What’s new?

- Our study provides the first long-term follow-up data on the use of Absorb BVS in a patient population reflecting daily clinical practice with regard to percutaneous coronary intervention.
- It is also the first study that reports long-term follow-up data without a previous intervention in dual anti-platelet therapy (DAPT) strategies (prolonging or re-starting).
- In this patient population, we found that cases of target-vessel failure continued to accrue beyond 2 years after Absorb BVS implantation.
- Moreover, we found 1 case of very late scaffold thrombosis that occurred 911 days after device implantation in a patient who was not on DAPT.

## Introduction

Coronary bioresorbable scaffolds have been developed to overcome the shortcomings of drug-eluting stents (DES). They are designed to provide temporary coronary scaffolding, in order to prevent acute recoil, and allow for vessel healing, and fully resorb over time [1]. The most widely used bioresorbable scaffold is the Absorb bioresorbable vascular scaffold (BVS) (Abbott Vascular, Santa Clara, CA, USA), which received CE approval in 2011 and FDA approval in 2016. Initial short-term results of studies conducted with the Absorb BVS were promising, with similar low rates of target-vessel failure (TVF) when compared to metallic DES [2]. Soon after the Absorb BVS became commercially available, the Amsterdam University Medical Centre—Academic Medical Centre (UMC-AMC) started to implant the Absorb BVS in a ‘real-world’ population, and started to follow these patients within the context of the AMC Single Centre Real World PCI Registry (hereafter referred as AMC Absorb Registry) [3]. The 2‑year results of the AMC Absorb Registry showed that the use of Absorb BVS in a patient registry reflecting daily clinical practice was associated with good procedural safety and acceptable clinical outcomes at mid-term (2-year) follow-up [4]. Higher rates of device thrombosis, however, have been reported up to 3 years after Absorb BVS implantation [5, 6]. Long-term follow-up after Absorb BVS implantation is therefore necessary in order to examine whether the annual event rates will decline after scaffold dismantling and resorption has been completed. In the current article, we therefore report long-term clinical outcomes of the AMC Absorb Registry.

### Methods

The design of the AMC Absorb Registry, the baseline, the procedural characteristics, the 6‑month clinical outcomes and the 2‑year clinical outcomes, have been reported previously [3, 4]. Briefly, in the AMC Absorb Registry, all patients who underwent percutaneous coronary intervention (PCI) with Absorb BVS implantation between 30 August 2012 and the 5 August 2013 at the Amsterdam UMC—AMC were included. The decision whether to implant the Absorb BVS was left to the discretion of the operator. We included patients with a wide range of indications, from presentation with stable angina pectoris to presentation with acute coronary syndrome (ACS). The necessity to obtain written informed consent from the included patients was waved by the institutional review committee. All patients received dual anti-platelet therapy (DAPT) for at least 12 months. Clinical follow-up was conducted through telephone contact, and if not possible by live status examination. All reported events were adjudicated by experienced interventional cardiologists (Y. Onuma (Erasmus MC, Rotterdam), P. Suwannasom (Cardialysis B.V., Rotterdam), and M. Beijk (Amsterdam UMC, Amsterdam)).

#### Definitions

The composite endpoint of this analysis was TVF, which was defined as a composite of cardiac death, target-vessel myocardial infarction (TV-MI) and target-vessel revascularisation (TVR). Secondary endpoints were MI, TVR, target-lesion revascularisation and scaffold thrombosis (ScT). All events were defined in accordance with the definitions of the Academic Research Consortium.

#### Statistical analysis

Continuous data are expressed as mean ± standard deviation or as median (interquartile ranges). Dichotomous data are summarised as frequencies (%). Cumulative event rates were estimated using the Kaplan-Meier method. All statistical analyses were performed with SPSS software, version 23 (IBM Corp., Armonk, NY, USA).

### Results

The median follow-up of the study cohort of the AMC Absorb Registry was 1534 days. At the time of the cross-sectional data sweep, the clinical status at 2 years was known in 132 of 135 (97.8%) patients, the clinical status at 3 years in 127 of 135 (94.1%) patients, and the clinical status at 4 years in 124 of 135 patients (91.9%). The baseline characteristics of the population are shown in Tab. 1. We enrolled 135 patients, in whom a total of 159 lesions were treated. Patients were predominantly male (73%); stable angina was the most common indication for PCI (47%). A total of 43 (40%) patients presented with ACS, of whom 17 (13%) presented with ST-elevation myocardial infarction.

**Table 1:** Baseline characteristics of the study population

*Patient characteristics*		*(n* *=135)*
Age (years)		59 ± 11
Male sex		98 (73%)
Diabetes mellitus		27 (20%)
Hypertension		67 (50%)
Hypercholesterolaemia		58 (43%)
Current smoker		39 (29%)
Renal dysfunction		11 (8%)
Previous myocardial infarction		34 (25%)
Previous PCI		35 (26%)
Previous CABG		3 (2%)
Multivessel disease		64 (47%)
Syntax Score		11.5 (IQ range: 6–17.5)
DAPT at discharge		135 (100%)
– Acetylsalicylic acid and clopidogrel		42 (31%)
– Acetylsalicylic acid and prasugrel		19 (14%)
– Acetylsalicylic acid and ticagrelor		74 (55%)
Indication for PCI	STEMI	17 (13%)
	NSTEMI	36 (27%)
	Unstable angina	13 (10%)
	Stable angina	63 (47%)
	Other	6 (4%)
*Lesion characteristics*		*(n* *=159)*
Vessels treated	LMCA	2 (1%)
	LAD	96 (60%)
	RCx	24 (15%)
	RCA	37 (23%)
Lesion type	A	27 (17%)
	B1	25 (16%)
	B2	67 (42%)
	C	40 (25%)
Bifurcation lesions		24 (15%)
Ostial lesions		5 (3%)
Calcified lesions		18 (11%)
Chronic total occlusion		13 (8%)
Thrombus present		14 (9%)

*PCI* percutaneous coronary intervention, *CABG* coronary artery bypass graft, *IQ* interquartile range, *DAPT* dual antiplatelet therapy, *STEMI* ST-elevation myocardial infarction, *NSTEMI* non-STEMI, *LMCA* left main coronary artery, *LAD* left anterior descending artery, *RCx* ramus circumflex artery, *RCA* right coronary artery

**Fig. 1:**
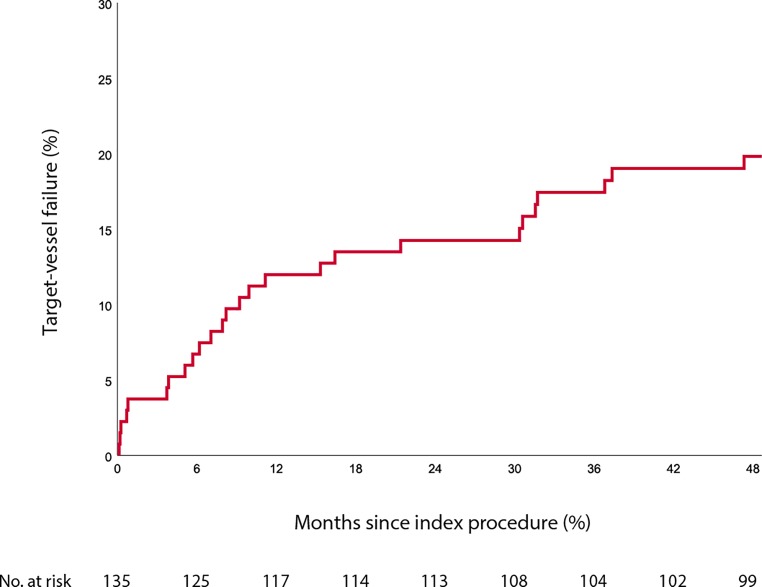
Kaplan-Meier curve of the outcome of target-vessel failure

An extensive description of the procedural and lesion characteristics has been published previously [7]. Briefly, most of the lesions treated with Absorb BVS (67%) were classified as type B2 or C (American Heart Association/American College of Cardiology classification); pre-dilatation was performed in 98% and post-dilatation in 55% of the treated lesions. The Syntax score ranged from 1 to 50, with a medium of 11.5 (interquartile range: 6–17.5). The clinical outcomes of all patients are shown in Tab. 2. At long-term follow-up, the composite endpoint of TVF had occurred in 27 patients. The 4‑year Kaplan-Meier estimate of TVF was 19.8% (Fig. 1). At 4 years cardiac death had occurred in 4 patients (3.2%) and TV-MI in 9 (6.9%) patients. Definite ScT had occurred in 5 (3.8%) patients. We found 1 case of very late ScT that occurred 911 days after device implantation in a patient who was not on DAPT. A detailed description of the cases with definite ScT is shown in Tab. 3. We found no cases of probable or possible device thrombosis.

**Table 2:** Long-term clinical outcomes of the study population

Outcome	Patients with an event	4‑year cumulative event rate
All-cause mortality	10	7.1%
Cardiac death	4	3.2%
Myocardial infarction	14	9.1%
Target-vessel myocardial infarction	9	6.9%
Target-vessel revascularisation	23	16.7%
Target-lesion revascularisation	18	14.6%
Definite scaffold thrombosis	5	3.8%
Probable/possible scaffold thrombosis	0	0%
Target-vessel failure	27	19.8%

**Table 3:** Detailed description of the cases of scaffold thrombosis

Case	Initial PCI indication	Treated vessel	Lesion type	Calcification	Pre-dilatation	Pre-dilatation balloon	Absorb size inflation pressure	Post-dilatation	Post-dilatation balloon	On DAPT	Timing (days)	Treatment	Possible reason
*1*	OHCA	Mid LAD	A	No	Yes	2.5 × 15 mm	3.0 × 18 mm (12)	Yes	2.5 × 15 mm NC (8)	Yes	4	Thrombus aspiration	Distal edge dissection
		Distal LAD	B2	No	Yes	2.5 × 15 mm	3.0 × 28 mm (14)	Yes	2.5 × 15 mm NC (10)			Xience 3.0 × 38 mm Xience 3.0 × 18 mm	
*2*	NSTEMI	Proximal LAD	B2	No	No	–	2.5 × 18 mm (16)	No	–	Yes	8	Thrombus aspiration Xience 2.5 × 28 mm	Incomplete expansion distal part of scaffold
*3*	Unstable AP	Distal LAD	B1	No	Yes	2.5 × 15 mm	2.5 × 28 mm (24)	Yes	2.5 × 15 mm NC (24)	No	24	Thrombus aspiration Xience 2.5 × 28 mm	DAPT cessation
*4*	NSTEMI	Proximal RCx	B1	No	Yes	2.5 × 15 mm	3.5 × 28 mm (10)	Yes	3.5 × 16 mm NC (16)	No	112	Thrombus aspiration multiple balloon inflations	DAPT cessation
*5*	Stable AP	Proximal RCA	A	No	Yes	2.5 × 15 mm	3.0 × 18 mm (10)	No	–	No	911	Azule 3.0 × 23 mm	DAPT cessation, intraluminal scaffold dismantling

*OHCA* out-of-hospital cardiac arrest, *LAD* left anterior descending artery, *NC* non-compliant, *NSTEMI* non-ST-elevation myocardial infarction, *AP* angina pectoris, *DAPT* dual antiplatelet therapy, *RCx* ramus circumflex artery, *RCA* right coronary artery

### Discussion

In the AMC Absorb Registry, the composite endpoint of TVF continued to accrue beyond 2 years after Absorb BVS implantation. Additionally, we found 1 case of very late ScT that occurred 911 days after device implantation in a patient who was not on DAPT. Absorb BVS was initially expected to be fully resorbed at 2 years after device implantation. Several studies have, however, reported that (very) late ScT also frequently occurs between the 2nd and 3rd year after device implantation, and even beyond 3 years, with the latest case of ScT reported as late as 44 months after Absorb BVS implantation [5, 6, 8, 9]. In these studies, the leading mechanisms of very late ScT were associated with (disintegrated) strut material that protruded into the coronary lumen, most likely as a consequence of intraluminal scaffold dismantling or late (acquired) malapposition [10]. DAPT is hypothesised to be an important factor in the prevention of thrombotic events after coronary stent implantation. When the preliminary safety report of the Amsterdam Investigator-initiateD Absorb Strategy All-comers (AIDA) trial (an international, multicentre, randomised trial in which the BVS was compared to DES) was published due to safety concerns regarding device thrombosis, the AIDA steering committee recommended cardiologists to consider re-starting or prolonging DAPT in patients treated with Absorb BVS up to 3 years after device implantation [11]. At that time point, all patients in the AMC Absorb Registry were already beyond 3 years after Absorb BVS implantation, so there was no additional medical intervention in this group, unlike in the AIDA trial. At 4‑year follow-up, in the AMC Absorb Registry, the estimated definite ScT rate was 3.8%, whereas in the comparable AIDA population the definitive ScT rate was 3.3% at 3 years. To date, it remains uncertain whether prolonged DAPT actually prevents (very late) ScT. Notably, in a large meta-analysis, 92% of the cases of very late ScT occurred in patients that were not on DAPT at the time of the event [12].

One of the causes hypothesised to be a (major) contributor to the adverse outcomes of Patients treated with Absorb BVS was suboptimal Absorb BVS implantation techniques. Optimised Absorb BVS implantation techniques, generally based on a pre-dilatation, sizing and post-dilatation (PSP) strategy were thereafter hypothesised to optimise outcomes after Absorb BVS implantation. The most correct definition of correct PSP implantation remains unknown, however, and its effect on the improvement of outcomes remains cumbersome and varies between studies [13–16]. Moreover, while suboptimal implantation techniques might explain (acute and early) events up to 3 years after implantation, it is difficult to stipulate that implantation techniques at the initial procedure impact outcomes at 4–5 years after device implantation, since the complex, and sometimes unpredictable and irregular, resorption process of the device occurs during this period.

In the ABSORB II trial, a downturn of events has been reported between the 3rd and 4th year of follow-up, thereby positively impacting the difference between the target-lesion failure rates of Absorb BVS and Xience EES at 4‑year follow-up [17]. Moreover, within the ABSORB III trial, in contrast to the pattern observed before 3 years, the event rates were similar between the Absorb BVS and Xience EES groups after 3 years [18]. The downturn in device-related events beyond 3 years after implantation in Absorb BVS patients within the ABSORB II and ABSORB III trials is encouraging. However, in the ABSORB II and ABSORB III trials, the patient populations were selected. In ABSORB II, patients that presented with an MI were excluded; and unstable patients and those with complex lesions were excluded from ABSORB III [19, 20].

Long-term follow-up, with precisely documented and investigated DAPT regimens/strategies, of large randomised studies such as the AIDA and the ABSORB IV trial, is therefore necessary in order to establish whether the annual rates of device-related-events in patients treated with Absorb BVS in routine clinical practice will decline after the period of complete scaffold dismantling and resorption.

#### Study limitations

This study is a registry, and therefore a control group is lacking. Second, the decision whether to implant an Absorb BVS was left to the discretion of the operator, and therefore potential patient selection bias has been introduced. Finally, routine intracoronary imaging has not been performed, and therefore potential information on the mechanism of scaffold failure might have been missed.

## Conclusion

In a patient population reflecting routine clinical practice, we found that cases of TVF continued to accrue beyond 2 years after Absorb BVS implantation.
